# Myocardial function at the early phase of traumatic brain injury: a prospective controlled study

**DOI:** 10.1186/s13049-016-0323-3

**Published:** 2016-10-28

**Authors:** Adrien Cuisinier, Claire Maufrais, Jean-François Payen, Stephane Nottin, Guillaume Walther, Pierre Bouzat

**Affiliations:** 1 Pôle Anesthésie Réanimation, Hôpital Albert Michallon, BP 217, Centre Hospitalier Universitaire de Grenoble, CS 10217, F-38043 Grenoble, France; 2 Laboratory of Integrative Cardiovascular and Metabolic Physiology, Division of Physiology, Department of Medicine, University of Fribourg, Fribourg, Switzerland; 3 Avignon University, LAPEC EA4278, F-84000 Avignon, France; 4 Grenoble Institut des Neurosciences, INSERM U1216, F-38043 Grenoble, France; 5 Grenoble Alpes Université, F-38043 Grenoble, France

## Abstract

**Background:**

The concept of brain-heart interaction has been described in several brain injuries. Traumatic brain injury (TBI) may also lead to cardiac dysfunction but evidences are mainly based upon experimental and clinical retrospective studies.

**Methods:**

We conducted a prospective case-control study in a level I trauma center. Twenty consecutive adult patients with severe TBI were matched according to age and gender with 20 control patients. The control group included adult patients undergoing a general anesthesia for a peripheral trauma surgery. Conventional and Speckle Tracking Echocardiography (STE) was performed within the first 24 post-traumatic hours in the TBI group and PRE/PER-operative in the control group. The primary endpoint was the left ventricle ejection fraction (LVEF) measured by the Simpson’s method. Secondary endpoints included the diastolic function and the STE analysis.

**Results:**

We found similar LVEF between the TBI group and the PER-operative control group (61 % [56–76]) vs. 62 % [52–70]). LV morphological parameters and the systolic function were also similar between the two groups. Regarding the diastolic function, the isovolumic relaxation time was significantly higher in the TBI cohort (125 s [84–178] versus 107 s [83–141], *p* = 0.04), suggesting a subclinical diastolic dysfunction. Using STE parameters, we observed a trend toward higher strains in the TBI group but only the apical circumferential strain and the basal rotation reached statistical significance. STE-derived parameters of the diastolic function tended to be lower in TBI patients.

**Discussion:**

No systematic myocardial depression was found in a cohort of severe TBI patients.

**Conclusions:**

STE revealed a correct adaptation of the left systolic function, while the diastolic function slightly impaired.

**Trial registration:**

NCT02380482

**Electronic supplementary material:**

The online version of this article (doi:10.1186/s13049-016-0323-3) contains supplementary material, which is available to authorized users.

## Background

Myocardial dysfunction has been described after brain injuries, leading to the concept of brain-heart interaction [1]. A paroxysmal sympathetic hyperactivity with higher plasma-level of endogenous norepinephrine has been observed after subarachnoid hemorrhage (SAH) and stroke, resulting in a cardiac dysfunction [2–4]. Transmural myocardial lesions were also reported after brain death in a series of post mortem examination [5]. Experimentally, the extreme elevation of intra-cranial pressure by inflating a subdural balloon induced acute cardiac failure related to an increased sympathetic activity, electrocardiographic abnormalities and myocardial damage [6, 7]. After traumatic brain injury (TBI), neurogenic pulmonary edema has been also reported [8] and high troponin concentrations were associated with an unfavorable neurologic outcome and mortality [9]. Recently, in a retrospective cohort of 139 patients with severe TBI, 12 % of the patients had a cardiac dysfunction with a reduced left ventricular ejection fraction (LVEF) and regional wall motion abnormalities [10]. These clinical findings were corroborated by experimental data which reported significant echocardiographic myocardial injury [11], while other authors reported no cardiac dysfunction within the first 24 h in a rat model of diffuse TBI [12]. Hence, the myocardial function after TBI remains unclear and its exploration relies upon experimental models and uncontrolled retrospective clinical studies. Possible confounding factors like associated multiple trauma, hemorrhagic shock, cardio-vascular co-morbidities or lung-heart interaction might also hinder the interpretation of the association between cardiac failure and severe TBI.

Assessment of cardiac function in clinical practice is based upon standard two dimensional (2D) echocardiography [13], that provides validated systolic and diastolic indices [14]. Since myocardial functionality results from a complex interplay between deformation (longitudinal, radial and circumferential) and twist/untwist mechanics, conventional echocardiography only partially described cardiac function. Conversely, Speckle Tracking Echocardiography (STE) offers a comprehensive and sensitive evaluation of regional myocardial function. For instance, in pathological conditions like sepsis or heart failure, conventional 2D-echocardiography demonstrated no LVEF impairment whereas speckle tracking echocardiography (STE) detected changes in myocardial function [15, 16].

The present study aimed at evaluating myocardial function at the early phase of TBI using conventional 2D echocardiography and complementary STE analysis. A matched cohort of non-TBI patients undergoing general anesthesia for a peripheral surgery served as control group. Our main hypothesis was a systematic 10 % decrease in the left ventricle ejection fraction in the TBI group compared to the control group.

## Methods

From November 2014 to November 2015, we conducted a prospective case-control single blinded study in a Level-I trauma center (Grenoble University Hospital, France). The regional ethics committee (number: 14-CHUG-34) and the national agency for drugs safety (Number: 2014-A01370-47) approved the study design. This trial was registered on ClinicalTrials.gov, number: NCT02380482. Written informed consent was obtained from the patient or patients’ next of kin.

### Patients

In the isolated TBI cohort, we included patients with a Glasgow Coma Score (GCS) lower than 9. Patients with a GCS from 9 to 13 were also included if mechanically ventilated due to severe cerebral injuries on computed tomography defined by a Marshall score from III to VI following the traumatic coma data bank (TCDB) [17]. We did not include patients with an expected brain death within 24 h and multiple trauma patients (Abbreviated Injury Score >2 in extra-cerebral region). In the control group, patients with a peripheral trauma undergoing a general anesthesia for an emergency surgery were included. Control patients were matched by gender and age (± 5 years) to the TBI patients. All patients (TBI and controls) were eligible if they were between 18 and 65 years old without cardiovascular risk factors. Exclusion criteria for both cohorts were: cardiovascular high-risk factors (diabetes, dyslipidemia or hypertension), a past medical history of cardio-thoracic surgery or significant cardiovascular events: stroke, myocardial infarction, congestive heart disease, pacemaker, atrial fibrillation, coronary artery disease, cardiac valvulopathy or implantable defibrillator. We also excluded patients with a traumatic hemorrhagic shock defined by a systolic arterial pressure below 90 mmHg, patients under inotrope therapy and elite athletes.

### Protocol

Screening and inclusions occurred 24/7 through a dedicated phone procedure within the study period. All patients who met the eligibility criteria were screened. In the TBI cohort, the echocardiography was performed at the intensive care unit (ICU) admission within the first 24 h after the trauma. In the control cohort, two ultrasound examinations were done. The first echocardiography (PRE operative) was performed before the general anesthesia under spontaneous ventilation. The second (PER operative) was performed just after the induction of general anesthesia under mechanical ventilation and before the start of surgery.

### Echocardiography image acquisition and analysis

Images were acquired by a certified operator (AC) in supine position, using ultrasound equipment (Vivid S5, GE Healthcare Company, USA) with a 2,5Mhz sector scanning electronic transducer. Images were obtained according to the standard and recommended procedure [14], and based on the average of three cardiac-cycles. Images were acquired for conventional 2D and strain acquisition (methodology is described in Additional file 1) in cine loops triggered to the QRS complex and were stored in a cine-loop format of 1 cardiac cycle of uncompressed data with associated electrocardiogram information. Each echocardiography was digitally recorded under a randomized and anonymous number. At the conclusion of the study a subsequent blinded off-line analysis for conventional and STE images was done, using a validated 2D STE tracking software (EchoPac 6.0, GE Healthcare, Horten, Norway). All ultrasound parameters were analyzed blindly to clinical data by AC and CM.

### Endpoints

The primary endpoint was the measurement of the left systolic function within the first 24 h after the trauma evaluated by the Simpson’s method.

Secondary endpoints were: 1) the left systolic function with the LVEF measured by the Teicholz method, the left ventricular shortening fraction, the peak velocity Sm, the cardiac output, the cardiac index and the stroke volume; 2) the left diastolic dysfunction assessed by the peak velocity of waves E, A, Em, ratio E/A, E/Em and IVRT; 3) the right function with the peak velocity S’m, the TAPSE and the pulmonary acceleration time; 4) STE variables for the LV evaluation: the global longitudinal strain (GLS) (%), the radial and circumferential peak strain (%) at the basal and apical level, the rotation (degree) at the basal and apical level, the peak twist (degree), twisting and untwisting velocities (degree/sec). Longitudinal, peak strain rate (%/sec), the rotational velocity (basal and apical level) (degree/sec) were measured separately for systole and diastole. The ratio between the untwisting velocity and the peak twist was also calculated.

### Data collection

For all patients, demographic data included age, gender, height, weight and body surface. The following hemodynamic and respiratory parameters were obtained during echocardiography examination: non-invasive blood pressure (systolic, mean and diastolic) with the corresponding dose of vasopressor, heart rate, fraction of inspired oxygen, positive end-expiratory pressure, respiratory rate, tidal volume per kilogram, type and doses of anesthetic drugs.

In the TBI cohort, we collected the last GCS before sedation, the TCDB classification and signs of elevated intracranial pressure (ICP) such as Cushing reflex, pupillary sizes, osmotherapy and seizure. Neurosurgical interventions were also reported: external ventricular drainage, craniotomy or decompressive craniectomy. The ICP value was collected when available. Biological data included troponin and NT pro-BNP. The electrocardiogram interpretation was done. The length of stay in the ICU, the duration of mechanical ventilation and the in-hospital mortality were also reported. In the non-TBI cohort, the reason for emergency surgery was collected.

### Sample size calculation

The study population size was calculated considering a LVEF of 65 (±5) % in the control group, as previously reported [18]. Assuming a clinically relevant reduction from 65 to 55 % in the TBI group, with a two-sided type 1 error of 0.05 and a power of 80 %, 20 patients per group were needed to detect this difference.

### Statistical analysis

Data were expressed as median and extreme. Univariate analysis between TBI group and control group was performed with the non-parametric Mann-Whitney test for continuous variables and with the chi-square for categorical variables. The statistical analysis was performed with StatView - SAS Institute Inc. 5.0 software. A *p* value < 0.05 was considered statically significant.

## Results

### Patients

Twenty consecutive TBI patients were included within the study period. Characteristics of the TBI patients are summarized in Table 1. The typical patient was young male suffering from a severe TBI. Fourteen patients had a GCS score lower than 9 and only six patients had a GCS between 9 and 13. All patients had severe lesions on the brain CT scan. Following the TCDB classification, five patients had diffuse injury and 15 patients had focal injury. Biological parameters were within normal ranges and ECG revealed no abnormality.

**Table 1:** Characteristics of patients with traumatic brain injury (*n* = 20 patients)

Variable	Value
Glasgow coma score	8 [3–13]
Anisocoric pupillary dilatation, n	3
Bilateral mydriasis, n	1
Cushing reflex, n	.3.
Osmotherapy, n	9
Seizure, n	4
External ventricular drainage, n	6
Immediate neurosurgery, n	6
Decompressive craniectomy, n	2
Norepinephrine, μg/kg/min	0.17 [0–0.47]
Troponin, μg/l	0 [0–0.42]
NT-ProBNP, mg/l	34 [15–1327]
ECG abnormalities, n	0
Diffuse injury (TCDB classification), n	
grade III	4
grade IV	1
Focal injury (TCDB Classification), n	
Surgical evacuation	6
No surgical evacuation but volume >25 ml	9
ICP value, mmHg	18 [3–50]
Length of stay in ICU, days	21 [3–52]
Duration of mechanical ventilation, days	13 [2–39]
In-hospital mortality, n	1

Values are presented as median [extreme] or number of patients. *TCDB* traumatic coma data bank, *ICP* intracranial pressure, *ICU* intensive care unit

Patients in the control group were admitted for an emergency surgery due to non-cerebral trauma: 11 patients for an orthopedic surgery, three patients for a maxillofacial surgery and six patients for a soft tissue surgery. There was no difference between patients with TBI and control patients, except for the respiratory rate and the volume per minute (Table 2). Other ventilation parameters and hemodynamic variables were similar between the TBI cohort and the control cohort. No patient in the control group received norepinephrine within the peri-operative period and no patient from the control group died within the study period. Sedation in the TBI group was maintained through propofol (*n* = 10 patients, dose = 5 [2–6] mg/kg/h) or midazolam (*n* = 10 patients, dose = 126 [84–270] μg/kg/h) infusions. All patients were sedated by an inhaled anesthesia in the control group (sevoflurane, *n* = 5 patients and desflurane, *n* = 15 patients) with a minimal alveolar concentration (MAC) value equal to 1 [0.4–1.2]. All patients received sufentanil for the pain management with a median dose equal to 0.48 [0.12–0.96] μg/kg/h. These findings indicated common doses for sedation and analgesia.

**Table 2:** Comparisons of main characteristics between Traumatic Brain Injury patients and control patients

Variable	TBI cohort *n* = 20 patients	Control cohort *n* = 20 patients
Age (years)	42 [19–57]	40 [18–62]
Male	17	17
Weight (kg)	72 [41–90]	75 [55–100]
Height (cm)	175 [160–185]	175 [165–185]
Body surface	1.9 [1.37–2.14]	1.91 [1.59–2.18]
Body mass index (kg/m^2^)	23.3 [16–27.7]	24.6 [20.1–32.7]
Mean Arterial Pressure (mmHg)	83 [68–101]	71 [55–120]
Heart rate (per minute)	70 [45–97]	74 [48–110]
Fraction of inspired oxygen (%)	40 [30–50]	45 [35–55]
Post End-Expiratory Pressure (cmH20)	5 [0–5]	5 [4–8]
Tidal Volume (ml)	465 [400–520]	450 [390–500]
Tidal Volume according to body weight (ml/kg)	6.5 [4.7–9.8]	6.1 [4.5–8]
Respiratory rate (cycles/per minute)	18 [13–22]	16 [12–18]*
Volume minute (L/min)	7.8 [6–10.4]	6.5 [5.6–8.1]*

Values are presented as median [extreme]. *kg* kilogram, *cm* centimeter, *Kg/m* ^*2*^ kilogram per square meter, *cmH20* centimeter of water, *mmHg* millimeter of mercury, *ml/kg* milliliter per kilogram, *L/min* liter per minute. *p < 0.05

### Primary outcome

**Fig. 1:**
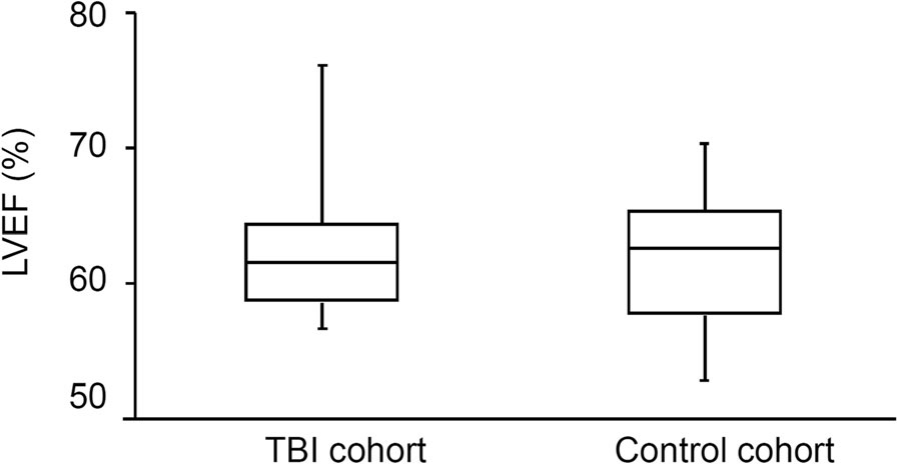
Left ventricle ejection fraction (LVEF) in patients with traumatic brain injury (TBI, *n* = 20 patients) and in control patients (*n* = 20 patients) obtained by conventional cardiac ultrasonography using the Simpson’s method. Echocardiography was performed within the first post-traumatic 24 h in the TBI group and under general anesthesia/mechanical ventilation in the control group. Values are median and extreme

The LVEF measured by the Simpson’s method was similar between the TBI cohort (61 % [56–76]) and the PER-operative control cohort (62 % [52–70], Fig. 1).

### Secondary outcomes

Conventional echocardiographic data of the TBI cohort and the PER-operative control cohort is presented on Table 3. LV morphological parameters and the systolic function were similar between the two groups. The IVRT was significantly higher in the TBI cohort. The peak E and E/A tended to be lower in TBI patients. The right ventricular systolic function was similar between the two groups.

**Table 3:** Univariate analysis between patients with TBI and control patients at the PER-operative phase regarding conventional echocardiography

Variables	TBI cohort *n* = 20 patients	PER-operative control *n* = 20 patients
Global systolic function		
LVEF (%) (Simpson biplan method)	61 [56–76]	62 [52–70]
Fractional shortening ratio (%)	37 [22–54]	38 [26–52]
LVEF (%) (Teicholz method)	65 [50–85]	69 [51–83]
Stroke volume (mL)	67 [41–103]	61 [46–71]
Cardiac output (L/min)	4.4 [2.7–7.3]	4.7 [2.6–6.5]
Peak Sm (cm/s)	13 [8–20]	12 [7–17]
Global diastolic function		
Peak E velocity (cm/s)	69 [44–100]	77 [56–106]
Peak A velocity (cm/s)	59 [29–90]	54 [30–109]
Peak E/A ratio	1.08 [0.67–2.44]	1.38 [0.84–2.40]
Peak Em (cm/s)	13 [6–24]	15 [9–24]
Isovolumic relaxation time (msec)	125 [84–178]	107 [83–141]*
E/Em ratio	4.8 [2.4–8.1]	5.3 [2.8–8.8]
Systemic vascular resistance index (dyn/s/cm5/m2)	2518 [1832–3955]	2341 [1030–3155]
Morphological parameters		
Left Atrial diameter (mm)	29 [19–36]	30 [23–36]
Left Ventricular end-diastolic volume (mL)	102 [66–156]	101 [82–132]
Left Ventricular end-systolic volume (mL)	37 [22–67]	39 [27–61]
Right ventricular diameter and function		
Right Ventricular end-diastolic diameter (mm)	22 [14–29]	23 [21–26]
Peak S’m (cm/s)	14 [8–19]	14 [11–18]
Tricuspid Annular Plane Systolic Excursion (mm)	23 [18–39]	24 [17–29]
Pulmonary acceleration time (msec)	152 [89–188]	138 [94–183]

Values are presented as median [extreme]. *TBI* traumatic brain injury, *mL* milliliter, *L/min* liter per minute, *L/min/m* ^*2*^ liter per minute per square meter, *cm/s* centimeter per second, *msec* millisecond, *dyn/s/cm5/m* ^*2*^ dynes per second per centimeter per square meter, *mm* millimeter. * *p* < 0.05

STE-derived parameters are presented in Table 4. We observed a trend toward higher strains in the TBI group but only the apical circumferential strain and the basal rotation reached statistical significance. STE-derived parameters of the diastolic function tended to be lower in TBI patients.

**Table 4:** Univariate analysis between TBI patients and control group at the PER-operative phase regarding Speckle Tracking Echocardiography

Variables	TBI cohort *n* = 20 patients	PER operative control *n* = 20 patients
Systolic function		
Global Longitudinal Strain	18 [10.3–23.6]	15.8 [12.4–22.5]
Circumferential Strain peak (%)		
basal level	17.2 [9.4–22.1]	16.4 [11.3–27.9]
apical level	26.9 [23.8–32.5]	23.2 [20.1–35.5]*
Radial Strain peak (%)		
basal level	14.8 [6–33.3]	13.1 [4.3–24.3]
apical level	16.3 [4.3–30.1]	13.9 [4.9–29.4]
Systolic longitudinal strain rate (%/s)	1.13 [0.73–1.52]	0.98 [0.73–1.33]
Rotation (deg)		
basal level	6.3 [2.4–10.8]	5 [1.5–10]*
apical level	7.29 [2.6–15]	8.89 [4.9–13.7]
Peak twist (deg)	12.6 [7.5–20.2]	12.8 [5.1–18.3]
Systolic rotational velocity (deg/s)		
basal level	66.3 [43.6–135.6]	64.7 [24.1–83.9]
apical level	62.2 [28.4–126.4]	73.8 [25.1–123.3]
Twisting velocity (deg/s)	77.2 [48.9–156.4]	76.6 [31.4–115]
Diastolic function		
Diastolic longitudinal strain rate (%/s)	1.33 [0.59–2]	1.56 [1.11–2.47]
Diastolic rotational velocity (deg/s)		
basal level	48.8 [22.3–97.5]	53.5 [18.5–156.5]
apical level	65.6 [31.6–109.6]	83.4 [35.6–112]
Untwisting velocity (deg/s)	91.6 [50.8–179.3]	100.4 [50.9–148.2]
Ratio: Untwisting velocity/Peak twist	7.73 [4–11.9]	7.92 [3.4–12.4]

Values are presented as median [extreme]. *TBI* trauma brain injury, *Deg* degree, *Deg/s* degree per second, *%/s* percentage per second. *p < 0.05

We found no difference in the control group between the PRE- and PER operative echocardiographic data (data available in the Additional file 2).

## Discussion

In this prospective controlled study, we did not find a systematic major myocardial dysfunction at the early phase of TBI with severe lesions on cerebral CT scan. These findings were obtained from conventional cardiac ultrasonography and speckle tracking analysis. Despite no major change in cardiac function, several STE changes suggested correct adaptation of the left systolic function and a slight impairment of the left diastolic function.

We reported the absence of alteration of the systolic function after a TBI since the LVEF was similar between the TBI and the control groups. More interestingly, none of the TBI patients had a decreased systolic function and the minimum LVEF was 56 % using the Simpson’s method. Speckle tracking analyses further confirmed these findings through similar systolic indices. Only the circumferential strain peak at the apical level and the rotation at the basal level were higher in the TBI cohort. These findings were in line with a trend toward a global increase of all systolic indices in STE. Taken together, these results rather suggested a slight increase in the systolic function after TBI since STE was able to detect early LV functional changes in the setting of systemic diseases with a cardiac issue [19]. Accordingly, a systemic adrenergic or sympathetic hyperactivity was described after TBI [20, 21]. We, thus, assume that these sympathetic and neuro-endocrine adaptations could be more responsible for an activation of systolic myocardial function rather than a global myocardial depression.

Another major result of the present study was the slightly impaired diastolic function. We only reported a significant higher IVRT in the TBI cohort. All STE-derived parameters tended to be lower in TBI patients without reaching a statistical significance. Taken together, we cannot conclude to any significant diastolic myocardial dysfunction at the early phase of TBI. However, the increase in the IVRT may be interpreted as a starting diastolic modification in the TBI cohort. A trend in lower diastolic STE indexes further confirmed this hypothesis. Similarly, diastolic dysfunction was previously described in patients with SAH and a concomitant pulmonary edema [22]. In a small series of seven patients with TBI, echocardiographic diastolic dysfunction was also reported in patients with a neurogenic pulmonary edema [8]. Taken together, these findings further corroborated the TBI-induced modifications in left diastolic parameters observed in our cohort. Exposition to vasopressor in the TBI cohort to maintain cerebral perfusion pressure may induce these modifications in our study. Even if our study was not designed to assess the effect of vasopressor on myocardial function, the use of vasopressor in the TBI cohort might account for an increase in left ventricle afterload with potential effects on systolic and diastolic parameters.

Our study is inconsistent with several experimental and clinical studies dealing with the brain-heart interaction. Several explanations may account for these controversial results. First, we investigated the myocardial function at the early phase of TBI, within the first 24 h. This time point was chosen according to the existing literature that described early myocardial dysfunction [23]. Accordingly, in a recent retrospective study, conventional cardiac ultrasonography was mostly performed on day one, revealing an acute myocardial depression after TBI [10]. Nevertheless, we cannot exclude a delayed myocardial dysfunction occurring within the first post-traumatic days [24]. In our study, slight systolic and diastolic STE changes might be undermined by the early ultrasound assessment and might be significant in later examinations. Second, we do not deny the concept of acute cardiac failure following brain injuries and the hemodynamic evaluation is part of the global assessment of severity in critically ill patients [25]. Nevertheless, the concept of neurogenic cardiac failure was mainly based on different experimental models [11] such as SAH [26] or brain death. In the clinical setting, echocardiography modifications [23] and electrocardiographic abnormalities [27] were also described in SAH patients, epileptic patients [28], stroke patients [24], or brain death patients [29]. Myocardial dysfunction after TBI has been extended from these conditions but only few studies showed an acute cardiac failure after TBI [10], mainly case report [30] and retrospective study [10]. Hence, there is no prospective controlled study comprehensively assessing cardiac function after TBI and possible confounding factors like multiple trauma, associated co-morbidities and hemorrhagic shock might induce a cardiac failure independently of the cerebral injury. To our knowledge, we conducted the first prospective controlled trial regarding cardiac dysfunction matching a TBI cohort with a control cohort according to age and gender. The potential sympathetic tonus due to emergency context and traumatic pathology was controlled since both cohorts were trauma patients admitted for an emergency treatment. Lung-heart interactions were also controlled since in both cohorts, patients were under mechanical ventilation and general anesthesia. We, thus, believe that our study adds to the existing literature to explore rigorously cardiac consequences of TBI.

We acknowledge several limits of our study. First, only two third of the TBI patients had a severe TBI according to the GCS definition. However, all TBI patients had severe injuries on cerebral CT scan. Six patients had a GCS between 9 and 13. Among them, three patients underwent an immediate neurosurgery, two patients had cerebral injuries classified TCDB VI and the last one had a compromised cerebral blood flow on the transcranial Doppler (Diastolic velocity = 26 cm/s and Pulsatility Index = 1,89). Despite relatively high GCS, these patients should be considered as severe from the clinical standpoint and did not explain the lack of difference between the TBI and the control groups. Moreover, the median length of stay in the ICU was 21 days with 13 days of mechanical ventilation attesting a global severity of the included patients. Second, we only tested the hypothesis of a systematic global decrease of the cardiac performance after TBI. We cannot conclude that a myocardial depression is not possible after TBI. We acknowledge that a neurogenic cardiac failure may occur in this context supporting the use of the conventional cardiac ultrasonography for hemodynamic management. Since TBI could be associated with preexisting comorbidities or a multiple trauma, we even strongly recommend the use of conventional ultrasonography at the bedside to optimize the cardiac output of these patients.

## Conclusions

Controlling confounding factors for non-cerebral acute cardiac failure, we did not find a systematic acute myocardial depression after TBI. STE analysis rather suggested an adequate adaptation of the left systolic function, while the diastolic function slightly decreased. The observed changes were not clinically relevant and our study further suggested no systematic cardiac consequences of severe traumatic cerebral injuries. These findings did not challenge the usefulness of cardiac ultrasonography for the management of TBI patients.

## Additional files

Additional file 1: Methodology of ultrasound acquisition. (DOCX 114 kb)
Additional file 2: Additional analyses. (DOC 161 kb)

## Abbreviations

2D: 2-dimensional
CVP: Central venous pressure
GLS: Global longitudinal strain
ICU: Intensive care unit
IVRT: Isovolumetric relaxation time
LA: Left atrial
LV: Left ventricle
LVEF: Left ventricular ejection fraction
MAC: Minimal alveolar concentration
MAP: Mean arterial pressure
RV: Right ventricle
SAH: Subarachnoid haemorrhage
STE: Speckle tracking echocardiography
SVRI: Systemic vascular resistance index
TAPSE: Tricuspid annular plane systolic excursion
TBI: Traumatic brain injury
TCDB: Traumatic coma data bank
