# Supplementary material for: Myocardial function at the early phase of traumatic brain injury: a prospective controlled study
Source: Scand J Trauma Resusc Emerg Med. 2016 Oct 28;24:129. doi: 10.1186/s13049-016-0323-3 (PMC5084439; doi:10.1186/s13049-016-0323-3)
Supplement: Additional file 1: — Methodology of ultrasound acquisition. (DOCX 114 kb) [file 13049_2016_323_MOESM1_ESM.docx]

**Supplemental data N°1: Methodology of ultrasound acquisition**

*Conventional 2D echocardiography*

In the apical four chamber view the LVEF was calculated from LV volumes measured by the biplane Simpson’s method. Pulsed Doppler LV trans-mitral velocities (Early (E), Atrial (A) waves) recordings were performed. The tricuspid annular plane systolic excursion (TAPSE) was measured to evaluate the right ventricle (RV) systolic function.

In the apical five-chamber view isovolumetric relaxation time (IVRT) was measured by pulsed Doppler, along with aortic ejection velocity. Stroke volume was calculated as the product of the aortic root area and the integral of the aortic blood flow velocity curve. Cardiac output was calculated as the product of the stroke volume and the heart rate. Cardiac index was calculated as the ratio between cardiac out put and body surface area. CVP (Central Venous Pressure) was estimated by measurement of the maximum diameter of the inferior cava vena. Systemic vascular resistance index (SVRI) was calculated (79.9 x (MBP-CVP)/cardiac index) (Stefadouros et al., 1973). In order to exclude an unknown cardiomyopathy or valvulopathy, a complete 2D examination was performed for all patients. Color flow Doppler valvular assessment was done.

In the parasternal long-axis view, M-Mode measurements were obtained. Left atrial (LA) diameter was measured at end diastole. The LV end-diastolic (EDD) and end-systolic (ESD) diameter, end-diastolic posterior wall thickness (EDPWT) and end-diastolic septum thickness (EDST) were measured. The left ventricle shortening fraction was obtained as the following ratio: [(LV EDD – LV ESD)/ LV EDD]. The RV end-diastolic diameter (EDD) was measured.

In the parasternal short-axis view pulmonary artery acceleration time was measured.

*Tissue Doppler imaging*

Wall motion was assessed by pulsed-TDI, with the measurement of the peak myocardial systolic velocity (Sm) and diastolic velocity (Em) at the mitral annulus level on the lateral wall in apical four chamber view. The *E/Em* ratio, recorded from the mitral annulus lateral wall, was used as an index of LV filling pressure (Nagueh et al., 2009). LV diastolic-dysfunction grading was determined using transmitral flow, and TDI indices, according to documented criteria (Nagueh et al., 2009)(Nagueh et al., 1997). Pulsed-TDI measure of myocardial systolic (S’m) velocity was assessed at the free wall of the RV at the tricuspid annulus.

*Speckle tracking echocardiography (STE)*

The STE acquisition and analysis was performed as previously described (Notomi et al., 2005)(Leitman et al., 2004)(Voigt et al., 2015) in order to quantify myocardial wall motion throughout a cardiac cycle. STE with strain (a measure of tissue deformation), SR (the rate at which deformation occurs) (Leung and Ng, 2010), rotation and twist/untwist velocities analysis were performed to investigate LV (Obert et al., 2012)(Crendal et al., 2013). On each cine loop, an optimal frame was selected with the best endocardial border definition. Strain was measured by tracing the endocardial border of the LV on the end systolic frame of 2D sequence across the maximal number of segments. LV longitudinal strain and SR were obtained in the apical 4-chamber view; LV radial and circumferential strains and SR were obtained in the parasternal short-axis view (at basal and apical levels of the LV). The software automatically tracked myocardial motion through an algorithm creating 6 equidistant speckles tracking regions of interest in each image and following endocardium. The average of these 6 regional values in the apical 4-chamber and parasternal basal short-axis views was used to measure longitudinal, radial, and circumferential strain and SR. All measurements for longitudinal, radial, and circumferential strain and SR were averages derived from three consecutive cardiac cycles, and were paired with real-time electrocardiogram. The tracking quality of all images was assessed before measurement and analysis. Images needed adequate endocardial border, frame rate and quality of tracking for being analyzed. In case of poor tracking efficiency, endocardial trace line and/or the region of interest could be readjusted by the observer. Inadequate images were excluded from strain measurements. LV rotations were assessed from basal and apical short axis views as average angular displacement of six myocardial segments. We ensured that the basal short-axis plane contained the mitral valve, and that the apical plane was acquired distally to the papillary muscle. LV peak twist was calculated as the maximal instantaneous difference between basal and apical rotations. LV twisting and untwisting velocities were calculated with specific software (Scilab 4.1, Consortium Scilab, INRIA-ENPC, Paris, France). These specific toolbox was used as well to adjust all STE imaging data between the subjects according to their heart rate, to normalize time sequence as a percentage of the systolic duration (Obert et al., 2012) (i.e aortic valve closure represented 100% of the systole duration).
