# Supplementary material for: Myocardial function at the early phase of traumatic brain injury: a prospective controlled study
Source: Scand J Trauma Resusc Emerg Med. 2016 Oct 28;24:129. doi: 10.1186/s13049-016-0323-3 (PMC5084439; doi:10.1186/s13049-016-0323-3)
Supplement: Additional file 2: — Additional analyses. (DOC 161 kb) [file 13049_2016_323_MOESM2_ESM.doc]

**Table 5.** Univariate analysis between patients with TBI and control patients at the PRE-operative phase regarding conventional echocardiography.

| **Variables** | **TBI cohort**  **n = 20 patients** | **PRE-operative control**  **n=20 patients** |
| --- | --- | --- |
| **Global systolic function** |  |  |
| Fractional shortening ratio (%) | 37 [22-54] | 40 [28-50] |
| LVEF (%) (Teicholz method) | 65 [50-85] | 71 [55-81] |
| Stroke volume (mL) | 67 [41-103] | 68 [56-85] |
| Cardiac output (L/min) | 4.4 [2.7-7.3] | 5 [2.8-8.6] |
| Cardiac index (L/min/m²) | 2.4 [1.5-3.6] | 2.6 [1.6-4.2] |
| Peak Sm (cm/s) | 13 [8-20] | 12 [8-19] |
| **Global diastolic function** |  |  |
| Peak E velocity (cm/s) | 69 [44-100] | 80 [55-100]* |
| Peak A velocity (cm/s) | 59 [29-90] | 63 [40-96] |
| Peak E/A ratio | 1.08 [0.67-2.44] | 1.19 [0.88-2.32] |
| Peak Em (cm/s) | 13 [6-24] | 16 [9-25] |
| Isovolumic relaxation time (msec) | 125 [84-178] | 118 [99-155] |
| E/Em ratio | 4.8 [2.4-8.1] | 5 [3.2-11] |
| Systemic vascular resistance index (dyn/s/cm5/m2) | 2518 [1832-3955] | 2359 [1370-4250] |
|  |  |  |
| **Morphological parameters** |  |  |
| Left Atrial diameter (mm) | 29 [19-36] | 34 [26-40]* |
| Left Ventricular end-diastolic volume (mL) | 102 [66-156] | 120 [74-155] |
| Left Ventricular end-systolic volume (mL) | 37 [22-67] | 43 [23-61] |
| Left Ventricular end-diastolic diameter (mm) | 44 [31-53] | 49 [39-54] |
| Left Ventricular end-systolic diameter (mm) | 28 [18-38] | 29 [22-35] |
| Left Ventricular End Diastolic Septal wall thickness (mm) | 10 [6-15] | 11 [7-17] |
| Left Ventricular End Diastolic Posterior wall thickness (mm) | 11 [7-13] | 10 [7-13] |
|  |  |  |
| **Right ventricular diameter and function** |  |  |
| Right Ventricular end-diastolic diameter (mm) | 22 [14-29] | 23 [20-26] |
| Peak S'm (cm/s) | 14 [8-19] | 15 [10-24] |
| Tricuspid Annular Plane Systolic Excursion (mm) | 23 [18-39] | 27 [20-33]* |
| Pulmonary acceleration time (msec) | 152 [89-188] | 131 [94-173]* |

Values are presented as median [extreme]. TBI: traumatic brain injury, mL: milliliter, L/min: liter per minute, L/min/m²: liter per minute per square meter, cm/s: centimeter per second, msec: millisecond, dyn/s/cm5/m²: dynes per second per centimeter per square meter, mm: millimeter. * p<0.05

**Table 6.** Univariate analysis between TBI patients and control group at the PRE-operative phase regarding Speckle Tracking Echocardiography.

| **Variables** | **TBI cohort**  **n = 20 patients** | | **PRE operative Control**  **n=20 patients** | |  |
| --- | --- | --- | --- | --- | --- |
| **Systolic function** | |  | |  | |
| Global Longitudinal Strain | | 18 [10.3-23.6] | | 17.8 [10-23.3] | |
| Circumferential Strain peak (%) | |  | |  | |
| basal level | | 17.2 [9.4-22.1] | | 17.7 [10.5-24.6] | |
| apical level | | 26.9 [23.8-32.5] | | 25.5 [17.7-33.6] | |
| Radial Strain peak (%) | |  | |  | |
| basal level | | 14.8 [6-33.3] | | 16.6 [6.8-34.7] | |
| apical level | | 16.3 [4.3-30.1] | | 15.3 [3.5-34.3] | |
| Systolic longitudinal strain rate (%/s) | | 1.13 [0.73-1.52] | | 0.97 [0.66-1.49] | |
| Systolic circumferential strain rate (%/s) | |  | |  | |
| basal level | | 1.24 [0.9-2.47] | | 1.15 [0.75-2.18] | |
| apical level | | 1.79 [1.09-2.39] | | 1.58 [0.94-2.68]* | |
| Systolic radial strain rate (%/s) | |  | |  | |
| basal level | | 1.09 [0.45-2.44] | | 1.37 [0.68-2.28] | |
| apical level | | 1.13 [0.42-2.41] | | 0.94 [0.49-1.99] | |
| Rotation (deg) | |  | |  | |
| basal level | | 6.3 [2.4-10.8] | | 5.1 [3.4-12] | |
| apical level | | 7.29 [2.6-15] | | 7.55 [3.36-12.8] | |
| Peak twist (deg) | | 12,6 [7.5-20.2] | | 14.2 [7.1-18] | |
| Systolic rotational velocity (deg/s) | |  | |  | |
| basal level | | 66.3 [43.6-135.6] | | 66.8 [31.4-140.9] | |
| apical level | | 62.2 [28.4-126.4] | | 52.4 [26.7-88.6] | |
| Twisting velocity (deg/s) | | 77.2 [48.9-156.4] | | 81.4 [40.5-170.9] | |
| **Diastolic function** | |  | |  | |
| Diastolic longitudinal strain rate (%/s) | | 1.33 [0.59-2] | | 1.47 [0.73-2.12] | |
| Diastolic circumferential strain rate (%/s) | |  | |  | |
| basal level | | 1.49 [0.65-2.66] | | 1.81 [0.42-3.15] | |
| apical level | | 1,93 [0.38-3.29] | | 2.02 [0.87-2.96] | |
| Diastolic radial strain rate (%/s) | |  | |  | |
| basal level | | 1.08 [0.39-2.79] | | 0.78 [0.38-2.4] | |
| apical level | | 1.04 [0.24-1.9] | | 1.28 [0.45-3.04] | |
| Diastolic rotational velocity (deg/s) | |  | |  | |
| basal level | | 48.8 [22.3-97.5] | | 57.1 [23.8-89.1] | |
| apical level | | 65.6 [31.6-109.6] | | 60.7 [32.1-98.6] | |
| Untwisting velocity (deg/s) | | 91.6 [50.8-179.3] | | 83.2 [61.9-147.4] | |
| Ratio : Untwisting velocity / Peak twist | | 7.73 [4-11.9] | | 6.56 [3.91-11.49] | |

Values are presented as median [extreme]. TBI: trauma brain injury, Deg: degree, Deg/s: degree per second, %/s: percentage per second. *p<0.05.

**Table 7.** Univariate analysis between control patients at the PRE-operative phase and control patients at the PER-operative phase regarding conventional echocardiography.

| **Variables** | **PRE-operative control**  **n = 20 patients** | **PER-operative control**  **n=20 patients** |
| --- | --- | --- |
| **Global systolic function** |  |  |
| Fractional shortening ratio (%) | 40 [28-50] | 38 [26-52] |
| LVEF (%) (Teicholz method) | 71 [55-81] | 69 [51-83] |
| Stroke volume (mL) | 68 [56-85] | 61 [46-71]* |
| Cardiac output (L/min) | 5 [2.8-8.6] | 4.7 [2.6-6.5] |
| Cardiac index (L/min/m²) | 2.6 [1.6-4.2] | 2.4 [1.5-3.6] |
| Peak Sm (cm/s) | 12 [8-19] | 12 [7-17] |
| **Global diastolic function** |  |  |
| Peak E velocity (cm/s) | 80 [55-100] | 77 [56-106] |
| Peak A velocity (cm/s) | 63 [40-96] | 54 [30-109] |
| Peak E/A ratio | 1.19 [0.88-2.32] | 1.38 [0.84-2.40] |
| Peak Em (cm/s) | 16 [9-25] | 15 [9-24] |
| Isovolumic relaxation time (msec) | 118 [99-155] | 107 [83-141] |
| E/Em ratio | 5 [3.2-11] | 5.3 [2.8-8.8] |
| Systemic vascular resistance index (dyn/s/cm5/m2) | 2359 [1370-4250] | 2341 [1030-3155] |
|  |  |  |
| **Morphological parameters** |  |  |
| Left Atrial diameter (mm) | 34 [26-40] | 30 [23-36]* |
| Left Ventricular end-diastolic volume (mL) | 120 [74-155] | 101 [82-132] |
| Left Ventricular end-systolic volume (mL) | 43 [23-61] | 39 [27-61] |
| Left Ventricular end-diastolic diameter (mm) | 49 [39-54] | 46 [37-52]* |
| Left Ventricular end-systolic diameter (mm) | 29 [22-35] | 29 [20-37] |
| Left Ventricular End Diastolic Septal wall thickness (mm) | 11 [7-17] | 10 [7-15] |
| Left Ventricular End Diastolic Posterior wall thickness (mm) | 10 [7-13] | 10 [8-13] |
|  |  |  |
| **Right ventricular diameter and function** |  |  |
| Right Ventricular end-diastolic diameter (mm) | 23 [20-26] | 23 [21-26] |
| Peak S'm (cm/s) | 15 [10-24] | 14 [11-18] |
| Tricuspid Annular Plane Systolic Excursion (mm) | 27 [20-33] | 24 [17-29]* |
| Pulmonary acceleration time (msec) | 131 [94-173] | 138 [94-183] |

Values are presented as median [extreme]. TBI: traumatic brain injury, mL: milliliter, L/min: liter per minute, L/min/m²: liter per minute per square meter, cm/s: centimeter per second, msec: millisecond, dyn/s/cm5/m²: dynes per second per centimeter per square meter, mm: millimeter. * p<0.05

**Table 8.** Univariate analysis between control group at the PRE-operative phase and control group at the PER-operative phase regarding Speckle Tracking Echocardiography.

| **Variables** | **PRE operative Control**  **n = 20 patients** | | **PER operative Control**  **n=20 patients** | |  |
| --- | --- | --- | --- | --- | --- |
| **Systolic function** | |  | |  | |
| Global Longitudinal Strain | | 17.8 [10-23.3] | | 15.8 [12.4-22.5] | |
| Circumferential Strain peak (%) | |  | |  | |
| basal level | | 17.7 [10.5-24.6] | | 16.4 [11.3-27.9] | |
| apical level | | 25.5 [17.7-33.6] | | 23.2 [20.1-35.5] | |
| Radial Strain peak (%) | |  | |  | |
| basal level | | 16.6 [6.8-34.7] | | 13.1 [4.3-24.3] | |
| apical level | | 15.3 [3.5-34.3] | | 13.9 [4.9-29.4] | |
| Systolic longitudinal strain rate (%/s) | | 0.97 [0.66-1.49] | | 0.98 [0.73-1.33] | |
| Systolic circumferential strain rate (%/s) | |  | |  | |
| basal level | | 1.15 [0.75-2.18] | | 1.21 [0.64-2.01] | |
| apical level | | 1.58 [0.94-2.68] | | 1.7 [0.83-2.22] | |
| Systolic radial strain rate (%/s) | |  | |  | |
| basal level | | 1.37 [0.68-2.28] | | 1.1 [0.5-2.41] | |
| apical level | | 0.94 [0.49-1.99] | | 0.9 [0.5-1.59] | |
| Rotation (deg) | |  | |  | |
| basal level | | 5.1 [3.4-12] | | 5 [1.5-10] | |
| apical level | | 7.55 [3.36-12.8] | | 8.89 [4.9-13.7] | |
| Peak twist (deg) | | 14.2 [7.1-18] | | 12.8 [5.1-18.3] | |
| Systolic rotational velocity (deg/s) | |  | |  | |
| basal level | | 66.8 [31.4-140.9] | | 64.7 [24.1-83.9] | |
| apical level | | 52.4 [26.7-88.6] | | 73.8 [25.1-123.3]* | |
| Twisting velocity (deg/s) | | 81.4 [40.5-170.9] | | 76.6 [31.4-115] | |
| **Diastolic function** | |  | |  | |
| Diastolic longitudinal strain rate (%/s) | | 1.47 [0.73-2.12] | | 1.56 [1.11-2.47] | |
| Diastolic circumferential strain rate (%/s) | |  | |  | |
| basal level | | 1.81 [0.42-3.15] | | 1.52 [1.06-2.79] | |
| apical level | | 2.02 [0.87-2.96] | | 2.12 [0.91-3.71] | |
| Diastolic radial strain rate (%/s) | |  | |  | |
| basal level | | 0.78 [0.38-2.4] | | 1.16 [0.5-2.1] | |
| apical level | | 1.28 [0.45-3.04] | | 1.16 [0.56-2.46] | |
| Diastolic rotational velocity (deg/s) | |  | |  | |
| basal level | | 57.1 [23.8-89.1] | | 53.5 [18.5-156.5] | |
| apical level | | 60.7 [32.1-98.6] | | 83.4 [35.6-112]* | |
| Untwisting velocity (deg/s) | | 83.2 [61.9-147.4] | | 100.4 [50.9-148.2] | |
| Ratio : Untwisting velocity / Peak twist | | 6.56 [3.91-11.49] | | 7.92 [3.4-12.4] | |

Values are presented as median [extreme]. TBI: trauma brain injury, Deg: degree, Deg/s: degree per second, %/s: percentage per second. *p<0.05.
